# ‘Porosome’ discovered nearly 20 years ago provides molecular insights into the kiss-and-run mechanism of cell secretion

**DOI:** 10.1111/jcmm.12598

**Published:** 2015-05-28

**Authors:** Bhanu P Jena

**Affiliations:** Department of Physiology, Wayne State University School of MedicineDetroit, MI, USA

**Keywords:** porosome, kiss-and-run, fusion pore, SNARE rosette, membrane fusion, ultra high-resolution atomic force microscopy, solution X-ray, mass spectrometry

## Abstract

Secretion is a fundamental cellular process in living organisms, from yeast to cells in humans. Since the 1950s, it was believed that secretory vesicles completely merged with the cell plasma membrane during secretion. While this may occur, the observation of partially empty vesicles in cells following secretion suggests the presence of an additional mechanism that allows partial discharge of intra-vesicular contents during secretion. This proposed mechanism requires the involvement of a plasma membrane structure called ‘porosome’, which serves to prevent the collapse of secretory vesicles, and to transiently fuse with the plasma membrane (*Kiss-and-run*), expel a portion of its contents and disengage. Porosomes are cup-shaped supramolecular lipoprotein structures at the cell plasma membrane ranging in size from 15 nm in neurons and astrocytes to 100–180 nm in endocrine and exocrine cells. Neuronal porosomes are composed of nearly 40 proteins. In comparison, the 120 nm nuclear pore complex is composed of >500 protein molecules. Elucidation of the porosome structure, its chemical composition and functional reconstitution into artificial lipid membrane, and the molecular assembly of membrane-associated t-SNARE and v-SNARE proteins in a ring or rosette complex resulting in the establishment of membrane continuity to form a *fusion pore* at the porosome base, has been demonstrated. Additionally, the molecular mechanism of secretory vesicle swelling, and its requirement for intra-vesicular content release during cell secretion has also been elucidated. Collectively, these observations provide a molecular understanding of cell secretion, resulting in a paradigm shift in our understanding of the secretory process.

- Introduction
- Discovery of the ‘Porosome’
  Membrane-associated t-SNAREs and v-SNARE in opposing bilayers interact in a rosette or ring complex, enabling Ca^2+^-mediated membrane fusion and the establishment of the ‘fusion pore’ at the porosome base
  Regulation of secretory vesicle volume increase and its requirement for fractional release of intra-vesicular contents from cells during secretion

## Introduction

**Figure 1:**
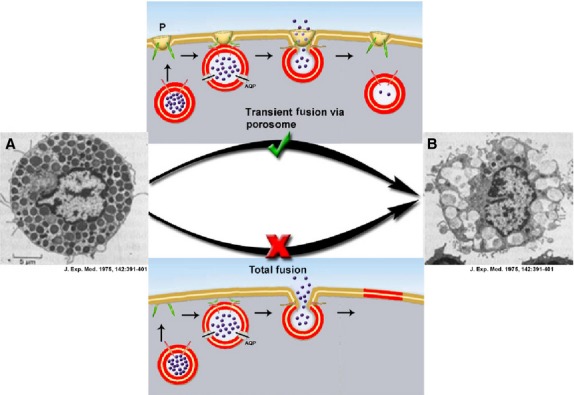
Electron micrographs of rat peritoneal mast cells in resting (A, extreme left) and following secretion (B, extreme right). Note the fractional release of intra-vesicular contents following secretion (B) (Electron micrographs obtained from *J. Exp. Med*. 142:391–401, 1975). This fractional release of intra-vesicular contents could only be possible *via* the porosome (P)-mediated transient fusion mechanism shown (√).

Secretion is a fundamental cellular process in living organisms, from yeast to cells in humans. Secretion is responsible and required for a variety of physiological activities, such as neurotransmission, immune response, and the release of hormones and digestive enzymes. Correspondingly, secretory defects in cells are responsible for a host of debilitating diseases. Since the mid 1950s, it was believed that secretory vesicles completely merge with the cell plasma membrane during secretion, resulting in release of the entire vesicular contents. While this provides one mechanism for cell secretion, the observation of partially empty vesicles in cells following a secretory episode (Fig.1) is incompatible with complete vesicle merger, suggesting the presence of an additional mechanism that allows partial discharge of intra-vesicular contents during secretion. This proposed mechanism requires the involvement of a plasma membrane structure the ‘porosome’, which serves to prevent the expected (because of high surface tension of the secretory vesicle membrane) complete collapse of secretory vesicles in the cell plasma membrane.

In the 1960s, the experimental data concerning neurotransmitter release mechanisms by Bernard Katz and Björn Folkow 1,2, proposed that *limitation of the quantal packet may be set by the nerve membrane, in which case the size of the packet released would correspond to a fraction of the vesicle content* 3,4. Then in the 1970s, Bruno Ceccarelli recognized the presence of ‘transient’ mechanism of secretory vesicle fusion at the cell plasma membrane 5 enabling the fractional release of intra-vesicular contents, and coined the term ‘kiss-and-run’. In 1990, Wolfhard Almers hypothesized based on his own and existing studies at the time, that the fusion pore is a continuity established between the vesicle membrane and the cell plasma membrane, and results from a ‘preassembled ion channel-like structure that could open and close’ 6. In a 1993 article 7, Erwin Neher appropriately reasoned that: ‘It seems terribly wasteful that, during the release of hormones and neurotransmitters from a cell, the membrane of a vesicle should merge with the plasma membrane to be retrieved for recycling only seconds or minutes later’. In an earlier 1992 article 8, Julio Fernandez opined that the principal difficulty in observing structures and fusion pore formation at these structures in the cell plasma membrane, was primarily due in part to the absence of ultra high-resolution imaging tools, to directly visualize and monitor the activity of such secretory portals in live cells. Such a membrane-associated portal would enable the secretory vesicle to transiently establish continuity with the cell plasma membrane without collapsing, expel a portion of the vesicular contents and disengage, while remaining partially filled as demonstrated in numerous cells, among them in rat peritoneal mast cells (Figs1 (√) and 2) and in acinar cells of the exocrine pancreas (Fig.3A and B).

**Figure 2:**
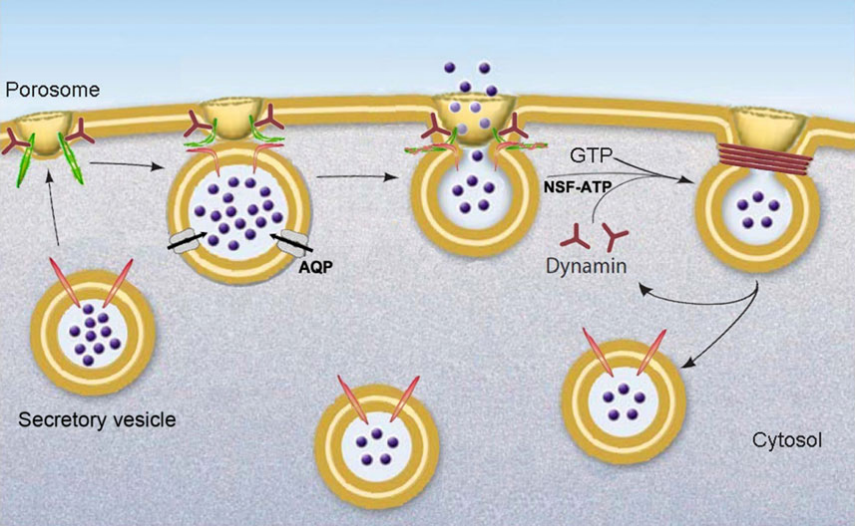
Schematic depiction of porosome-mediated cell secretion. Porosomes are cup-shaped lipoprotein structures at the cell plasma membrane. Secretory vesicles dock at the porosome base *via* t-SNAREs present at the porosome base and v-SNAREs present at the secretory vesicle membrane to form a *t-/v-SNARE ring complex*, establishing continuity between the opposing membranes [*fusion pore*] through which pressurized intra-vesicular contents (intra-vesicular pressure established *via* active transport of water through aquaporin or water channels (AQP), and ions at the secretory vesicle membrane) are expelled to the outside during cell secretion. Following secretion, the SNARE ring complex is disassembled by NSF-ATP and the fused lipid membrane is cleaved by dynamin-GTP. The resultant partially empty vesicle then dissociates from the cell membrane, either to be refilled (in case of synaptic vesicles containing neurotransmitter transporters at the vesicle membrane), reused in one or multiple exo-endocytosis until empty, or recycled to generate new secretory vesicles. ©Bhanu Jena.

**Figure 3:**
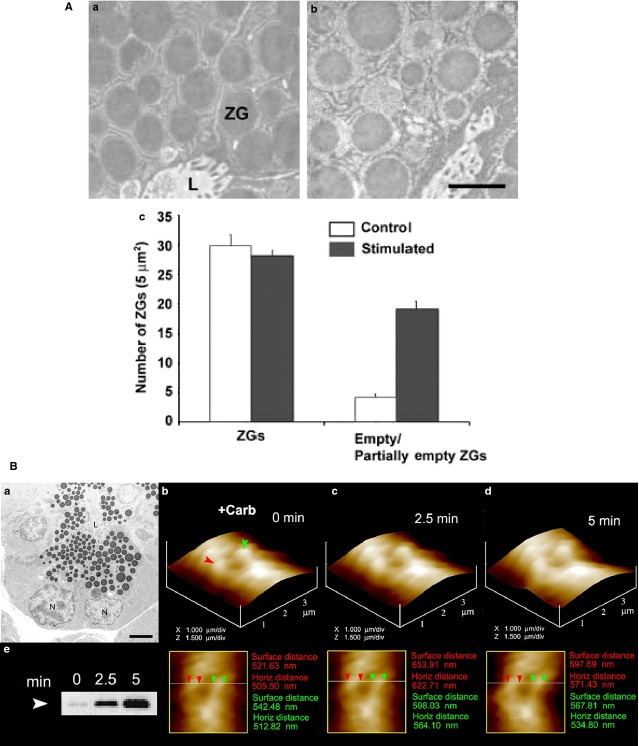
(A) Representative electron micrographs of resting (a), and 1 μM cholecystokinin-stimulated for 15 min. (b) rat pancreatic acinar cells, demonstrating partial loss of zymogen granule (ZG) contents following secretion. The apical lumen (L) of acini demonstrating the presence of microvilli and secreted products is observed. (c) These studies using electron microscopy further demonstrate that while the number of ZG remain unchanged following secretion, an increase in the number of empty and partially empty vesicles are observed; scale bar = 1 μm 10. ©Bhanu Jena. (B) The volume dynamics of zymogen granules (ZG) in live pancreatic acinar cells demonstrating fractional release of ZG contents during secretion. (a) Electron micrograph of pancreatic acinar cells showing the basolaterally located nucleus (N) and the apically located electron-dense vesicles, the ZGs. The apical end of the cell faces the acinar lumen (L); bar = 2.5 μm. (b–d) Apical ends of live pancreatic acinar cells in physiological buffer imaged by AFM, showing ZGs (red and green arrowheads) lying just below the apical plasma membrane. Exposure of the cell to a secretory stimulus (1 μM carbamylcholine), results in ZG swelling within 2.5 min., followed by a decrease in ZG size after 5 min. The decrease in size of ZGs after 5 min. is because of the release of secretory products such as α-amylase, as demonstrated by the immunoblot assay (e). If ZG’s had fused at the plasma membrane and fully merged, it would not be visible, demonstrating transient fusion and fractional discharge on intra-vesicular contents during secretion in pancreatic acinar cells 32. ©Bhanu Jena.

In acinar cells of the exocrine pancreas, partially filled zymogen granules (ZG), the secretory vesicles in these cells, are generated following a secretory episode as observed in electron micrographs (EM; Fig.3A). Electron micrographs morphometry of intracellularly located ZG in pancreatic acinar cells demonstrate that although the total number of ZG in cells remain unchanged following secretion, there is an increase in the number of empty and partially empty vesicles following a secretory episode, suggesting a transient kiss-and-run mechanism of intra-vesicular content release during cell secretion (Fig.3A and B). Further confirmation of the transient or kiss-and-run mechanism of cell secretion is demonstrated by the direct observation using atomic force microscopy (AFM) of docked ZG at the apical plasma membrane in live pancreatic acinar cells. Physiological stimulation of cell secretion using the CCK analogue carbamylcholine results first in ZG swelling (a requirement for cell secretion), followed by intra-vesicular content release and a consequent decrease in ZG size; however, the same ZG’s remain, long after the completion of the secretory episode (Fig.3B), demonstrating the occurrence of transient or kiss-and-run mechanism of cell secretion.

So what is this structure that enables transient secretory vesicle fusion and the regulated fractional release of intra-vesicular contents from cells during secretion? This structure at the membrane needs to overcome the surface tension of the vesicle membrane and prevent collapse of the vesicle at the cell plasma membrane. This conundrum was finally resolved in 1996 following the discovery of the ‘porosome’, a supramolecular lipoprotein structure at the cell plasma membrane, which has since been demonstrated to be ubiquitously found in all cells examined, and hence defined as the universal secretory portal in cells 6. Porosomes are cup-shaped lipoprotein structures at the cell plasma membrane, where membrane-bound secretory vesicles transiently dock and fuse to release intra-vesicular contents to the outside during cell secretion. Almost two decades ago, using the AFM, followed by EM and other imaging modalities like small angle X-ray solution scattering (SAXS), this cup-shaped plasma membrane lipoprotein structure initially misnamed ‘fusion pore’ was subsequently renamed ‘porosome’. Fusion pore is the continuity established between two fusing membranes, and develops at the porosome base when the secretory vesicle membrane fuses. During secretion, secretory vesicles transient dock and fuse at the base of the porosome cup *via* SNAREs to establish such a fusion pore or continuity between the secretory vesicle membrane and the porosome base. To enable precisely measured release of intra-vesicular contents, immediately prior to vesicle fusion at the porosome, secretory vesicles swell *via* regulated active transport of water and ions, and the resultant intra-vesicular pressure generated drives the intra-vesicular contents to the outside, without compromising the integrity of either the vesicle membrane or the cell plasma membrane 9–17.

Meanwhile, in the past 20 years, in further confirmation, hundreds of papers from scores of laboratories from around the world provide evidence on the kiss-and-run mechanism of cell secretion and fractional discharge of intra-vesicular contents from cells. Among these publications on porosomes and on the consequent kiss-and-run-mediated process of cell secretion, it has been demonstrated that ‘secretory granules are recaptured largely intact following stimulated exocytosis in cultured endocrine cells’ 18; ‘single synaptic vesicles fuse transiently and successively without loss of identity’ 19; and ‘zymogen granule exocytosis is characterized by long fusion pore openings and preservation of vesicle lipid identity’ 20. Utilizing the porosome-mediated kiss-and-run mechanism of secretion in cells, secretory vesicles are capable of being reused for subsequent rounds of exo-endocytosis, until completely empty of contents. However, in a fast secretory cell such as the neuron, synaptic vesicles have the additional advantage of rapidly refilling, utilizing the neurotransmitter transporters present at the synaptic vesicle membrane.

Elucidation of the porosome structure, its chemical composition and functional reconstitution into artificial lipid membrane 9–17, and the molecular assembly of membrane-associated t-SNARE and v-SNARE proteins in a ring or rosette complex 21–29, resulting in the establishment of membrane continuity between the membrane of the porosome base and the secretory vesicle membrane to establish a *fusion pore*, has also been demonstrated in great detail in the past two decades 21–29. Furthermore, the molecular mechanism of secretory vesicle swelling and its requirement for the precise regulation of intra-vesicular content release during cell secretion has been determined 30–36. Collectively, these studies provide a molecular understanding of porosome-mediated kiss-and-run mechanism of fractional discharge of intra-vesicular contents from cells during secretion, resulting in a paradigm-shift in our understanding of the secretory process.

## Discovery of the ‘Porosome’

**Figure 4:**
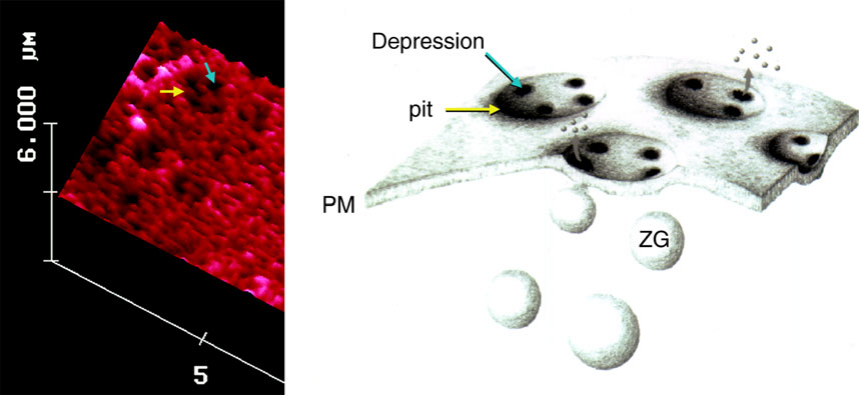
To the left is an AFM micrograph of the apical plasma membrane of a live pancreatic acinar cell demonstrating the presence of a pit (yellow arrow) with porosomes within (blue arrow). To the right is a schematic drawing demonstrating pits and cup-shaped porosomes where zymogen granules (ZG), the secretory vesicles in exocrine pancreas dock and transiently fuse to release intra-vesicular digestive enzymes from the cell 9. ©Bhanu Jena.

**Figure 5:**
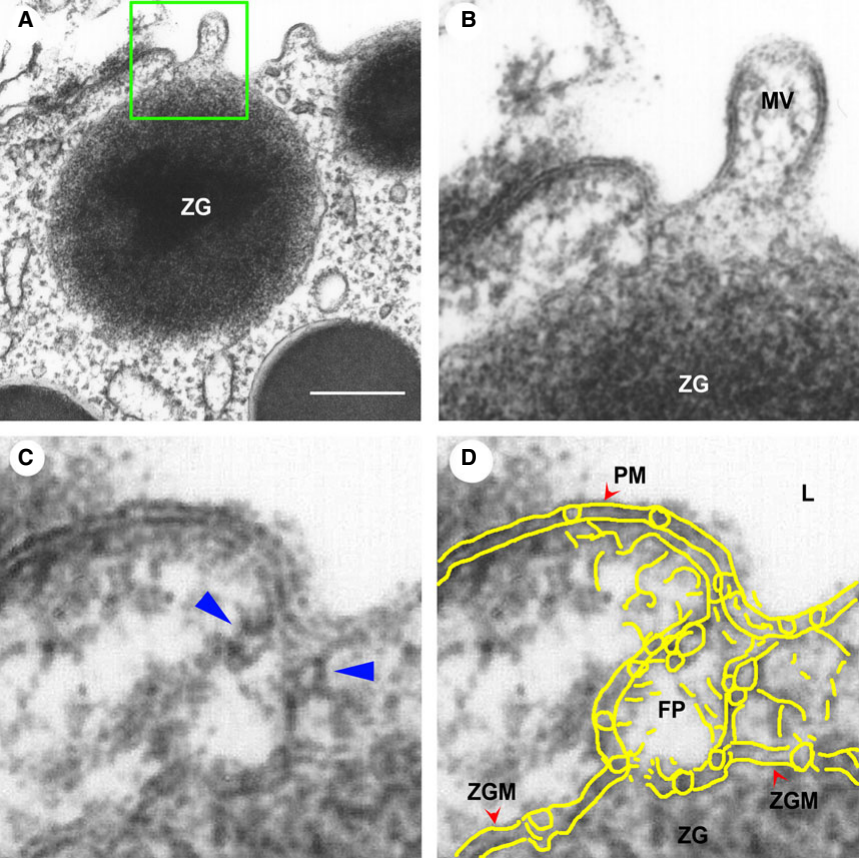
Transmission electron micrograph of a porosome associated with a docked secretory vesicle at the apical end of a pancreatic acinar cell. (A) Part of the apical end of a pancreatic acinar cell demonstrating within the green square, the presence of a porosome and an associated zymogen granule (ZG) fused at its base. (bar = 400 nm only in A). (B) The area within the green square in A, has been enlarged to show the apical microvilli (MV) and a section through the porosome and the ZG. Note the ZG membrane (ZGM) bilayer is fused at the base of the porosome cup. (C) A higher magnification the porosome-associated ZG shows in greater detail the porosome bilayer and cross-section through the three protein rings (which appear as knobs in either side of the cup-shaped porosome), with the thicker ring (blue arrowhead) present close to the opening of the porosome to the outside, which may regulate the closing and opening of the structure. The third and the lowest ring away from the porosome opening is attached to the ZGM, and may represent the t-/v-SNARE rosette or ring complex. (D) Yellow outline of the ZG fused porosome complex (FP) demonstrating the continuity with the apical plasma membrane (PM) at the apical end of the pancreatic acinar cell facing the lumen (L). The exact points of contact and fusion of the ZGM with the membrane at the porosome base is clearly seen in the micrograph. 14. ©Bhanu Jena.

In the mid 1990s, motivated by the goal to identify cellular structures at the plasma membrane involved in the regulated fractional release of intra-vesicular contents from cells, the newly developed technique of AFM was employed by my group to image the morphology and dynamics of the live pancreatic acinar cell surface at the nanometre scale resolution in real-time during secretion. Utilizing this approach, the major breakthrough came in 1996, when circular pit-like structures containing 100–180 nm depressions or pores were observed at the apical plasma membrane of live pancreatic acinar cells (Figs4 and 5), where secretion is known to occur. During secretion, the depressions or pores grew larger, returning to their resting size following completion of cell secretion. These results were first reported online ahead of print in 1996 and then in print in the January 1, 1997 issue of the Proceedings of the National Academy of Sciences, USA 9. After 5 years of careful study, our results finally confirmed that the 100–180 nm depressions or pores at the apical plasma membrane of pancreatic acinar cells are secretory portals 10,11, where secretory vesicles transiently dock and fuse to expel intra-vesicular contents to the outside during cell secretion. In January 2002 and February 2003, we reported in two studies, one in Cell Biology International in collaboration with Douglas J. Taatjes 10 and the other in the Biophysical Journal 11, that following stimulation of cell secretion, gold-conjugated amylase antibodies (amylase being one of the major intra-vesicular enzymes secreted by the exocrine pancreas) accumulate at depressions, establishing that depressions are the long sought-after secretory portals in cells 10,11. Our studies reported in the Biophysical Journal 11, further demonstrated using immuno-AFM, the presence of t-SNAREs at the porosome base facing the cytosol, firmly establishing depression structures to be secretory portals where ZG transiently dock and fuse for intra-vesicular content release during secretion 11. In March of 2002, our laboratory in collaboration with Lloyd L. Anderson reported in the journal Endocrinology 12 on the depressions and their dynamics at the cell plasma membrane in growth hormone (GH) secreting cells of the pig pituitary gland and on the accumulation of GH-immuno-gold at depressions following GH secretion from these cells. In the same year (2002) in a separate study, in collaboration with Arun Wakade and George D. Pappas, our group reported depression structure and their dynamics in rat chromaffin cells 13. In September of 2003 14 following immunoisolation of the depression structures from acinar cells of the exocrine pancreas, our team finally determined their composition, and we succeeded in functionally reconstituting the isolated porosome complex into artificial lipid membranes 14 using the EPC9 electrophysiological bilayer set-up originally developed in the laboratory of Erwin Neher. In the same study 14, morphological details of the porosome complex associated with docked secretory vesicle with established fusion pore was revealed at ultra high-resolution using electron microscopy (EM) 14 (Fig.5).

**Figure 6:**
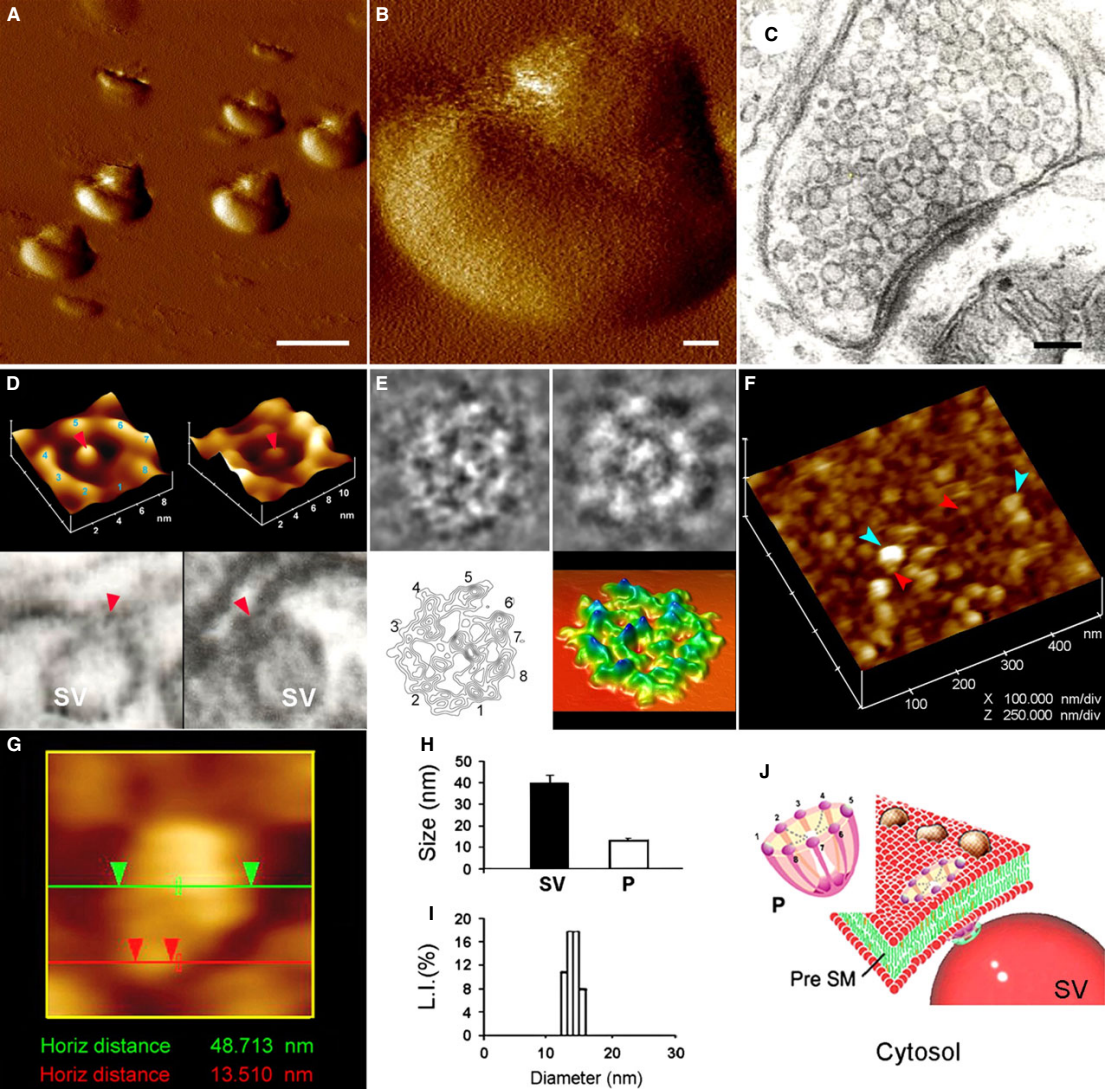
Structure and organization of the neuronal porosome complex at the nerve terminal 15,16. (A) Low-resolution AFM amplitude image bar = 1 μm (A) and high-resolution AFM amplitude image bar = 100 nm (B) of isolated rat brain synaptosomes in buffered solution. (C) Electron micrograph of a synaptosome, bar = 100 nm. (D) Structure and arrangement of the neuronal porosome complex facing the outside (D top left), and the arrangement of the reconstituted complex in PC:PS membrane (D top right). Lower panels are two transmission electron micrographs demonstrating synaptic vesicles (SV) docked at the base of cup-shaped porosome, having a central plug (red arrowhead). (E) EM, electron density, and 3D contour mapping (E), provides at the nanoscale, the structure and assembly of proteins within the complex. (F) AFM micrograph of inside-out membrane preparations of isolated synaptosome. Note the porosomes (red arrowheads) to which synaptic vesicles are found docked (blue arrow head). (G) High-resolution AFM micrograph of a synaptic vesicle docked to a porosome at the cytoplasmic compartment of the presynaptic membrane. (H) AFM measurements (n = 15) of porosomes (P, 13.05 ± 0.91) and SV (40.15 ± 3.14) at the cytoplasmic compartment of the presynaptic membrane. (I) Photon correlation spectroscopy (PCS) on immunoisolated neuronal porosome complex demonstrates their size to range from 12 to 16 nm. (J) Schematic illustration of a neuronal porosome at the presynaptic membrane, showing the eight peripheral ridges connected to the central plug 15,16. ©Bhanu Jena.

In 2004, the neuronal porosome complex was first discovered by our group, isolated and functionally reconstituted into artificial lipid membrane using the EPC9 bilayer set-up 15. In 2012, the proteome of the neuronal porosome complex 16, and in 2014, its lipidome was finally determined 38. Examination of the presynaptic membrane at the nerve terminal using high-resolution AFM 15, EM 15 and SAXS studies 17 demonstrate the presence of approximately 15 nm cup-shaped porosomes, each possessing a central plug (Figs8). The outer rim of the porosome opening to the outside is lined by eight equally spaced protein densities (Fig.6D and E). The eight protein densities are observed both in the native neuronal porosome complex (Fig.6D top left) as well as in the isolated porosomes reconstituted in lipid bilayers prepared using brain phosphatidylethanolamine, phosphatidylcholine (PC), dioleoylphosphatidylcholine and dioleoylphosphatidylserine (Fig.6D top right). Similar to AFM micrographs, approximately eight interconnected protein densities are observed in EM micrographs of purified neuronal porosome preparations (Fig.6E). Electron density and contour mapping, and the resultant three-dimensional (3D) topology profiles of the neuronal porosome complex provide further details of the arrangement of proteins and their interconnection to the central plug region of the complex *via* distinct spoke-like elements (Fig.6E lower left). The 3D topography of the porosome complex (Fig.6E lower right) obtained from electron density maps, show in further detail, the circular profile of the porosome complex and a central plug connected *via* spokes as in a cart wheel. Atomic force microscopy micrographs of inside-out presynaptic membrane demonstrate inverted cup-shaped porosomes (facing the cytosolic compartment), some with synaptic vesicles docked to the porosome base (Fig.6F and G). Atomic force microscopy, EM and photon correlation spectroscopy (Fig.6H and I), all demonstrate isolated porosomes that range in size from 12 to 17 nm. Interestingly, high-resolution AFM micrographs of the neuronal porosome complex present at the presynaptic membrane demonstrates the central plug at various conformation states, such as fully distended at an intermediate position, and sometimes completely withdrawn into the porosome cup suggesting the capability of the central plug for vertical motion and its possible involvement in the rapid opening and closing of the structure. Besides ultra high-resolution AFM studies on isolated synaptosomes revealing structural details of the neuronal porosome complex, the porosome complexed with synaptic vesicle in its native state *in situ* within synaptosomes, has also been examined using SAXS providing further molecular details of the complex and its interaction with synaptic vesicles 17. Results from the SAXS study has enabled the development of a model on the possible involvement of the central plug in neurotransmitter release at the nerve terminal.

**Figure 7:**
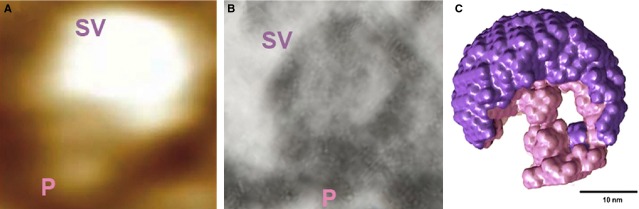
Docked synaptic vesicles at neuronal porosome complex in the presynaptic membrane of the nerve terminal, observed using atomic force microscopy (AFM), electron microscopy (EM) and small angle X-ray solution scattering (SAXS). (A) AFM micrograph obtained in fluid of a synaptic vesicle (SV) docked at the cup-shaped porosome complex (P) at the cytosolic compartment of the presynaptic membrane. Note the 35 nm SV docked to a 15 nm porosome complex. (B) An EM micrograph of a 35 nm SV docked to a 15 nm P at the presynaptic membrane 15. Note the central plug of the porosome complex in the electron micrograph. (C) The averaged SAXS 3-D structure of synaptic vesicle (purple) docked at the cup-shaped neuronal porosome complex (pink) at the presynaptic membrane in isolated synaptosomes, is presented 17. Note that AFM, EM and SAXS, all demonstrating similarity in the docking and interaction of synaptic vesicles with the neuronal porosome complex at the presynaptic membrane. ©Bhanu Jena.

**Figure 8:**
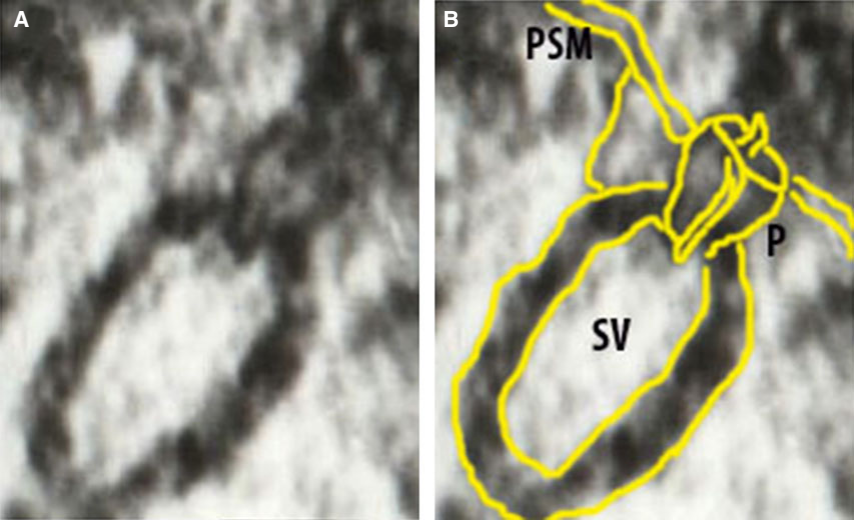
Electron micrograph of a docked synaptic vesicle (SV) at the base of a cup-shaped neuronal porosome complex (P), present at the presynaptic membrane (PSM) of a nerve terminal in a rat brain neuron. (A) A 50 nm synaptic vesicle docked at the base of a 15 nm porosome complex. Scale bar = 10 nm. Note the central plug at the porosome complex. (B) The synaptic vesicle, the porosome complex with a central plug, and the presynaptic membrane, are outlined in yellow for clarity. *Micron* (2012) 43:948-953. *Courtesy of M. Zhvania*.

In the past 20 years, our studies demonstrate that membrane-associated t-SNAREs and v-SNARE interact in a rosette or ring complex, enabling Ca^2+^-mediated membrane fusion and establishment of the ‘fusion pore’ 21–29. Furthermore, our studies have progressed our understanding of the regulation of secretory vesicle volume and the requirement of vesicle volume increase for fractional release of intra-vesicular contents from cells during secretion 30–36. These results provide the molecular underpinnings of how cells precisely regulate the discharge of a portion of their intra-vesicular contents during a secretory episode, while retaining full integrity of both the vesicle membrane and the cell plasma membrane.

### Membrane-associated t-SNAREs and v-SNARE in opposing bilayers interact in a rosette or ring complex, enabling Ca^2+^-mediated membrane fusion and the establishment of the ‘fusion pore’ at the porosome base

In 1988, Richard Scheller discovered a secretory vesicle associated membrane protein called VAMP-1 or v-SNARE 39, and then in 1992, he and his team discovered another important protein present in the cell plasma membrane called syntaxin. Syntaxin is one of the two target SNARE or t-SNARE proteins 40. In 1989, Michael Wilson discovered SNAP-25, the other t-SNARE protein 41. Understanding the properties of the three SNARE proteins in membrane fusion requires a molecular understanding of their interactions, with the different SNARE proteins being present in opposing membranes: the v-SNARE or VAMP-1 in secretory vesicle membrane, and t-SNAREs syntaxin and SNAP-25 in the cell plasma membrane. As SNAREs are membrane-associated proteins, crystals of membrane-associated SNARE complex are required for X-ray crystallography, which has not been possible. To circumvent issues associated with the solubility of membrane-associated SNAREs, Axel Brunger and Reinhard Jahn in 1998 truncated the hydrophobic membrane anchoring domains of syntaxin and VAMP, to obtain crystals of a non-membrane-associated t-/v-SNARE complex. Utilizing X-ray crystallography, Brunger and Jahn determined the atomic structure of the soluble SNARE complex at 2.4 Å, which they reported in Nature 42. It was unclear, however, whether the structure of the resolved soluble SNARE complex was identical to the native membrane-associated SNARE complex.

**Figure 9:**
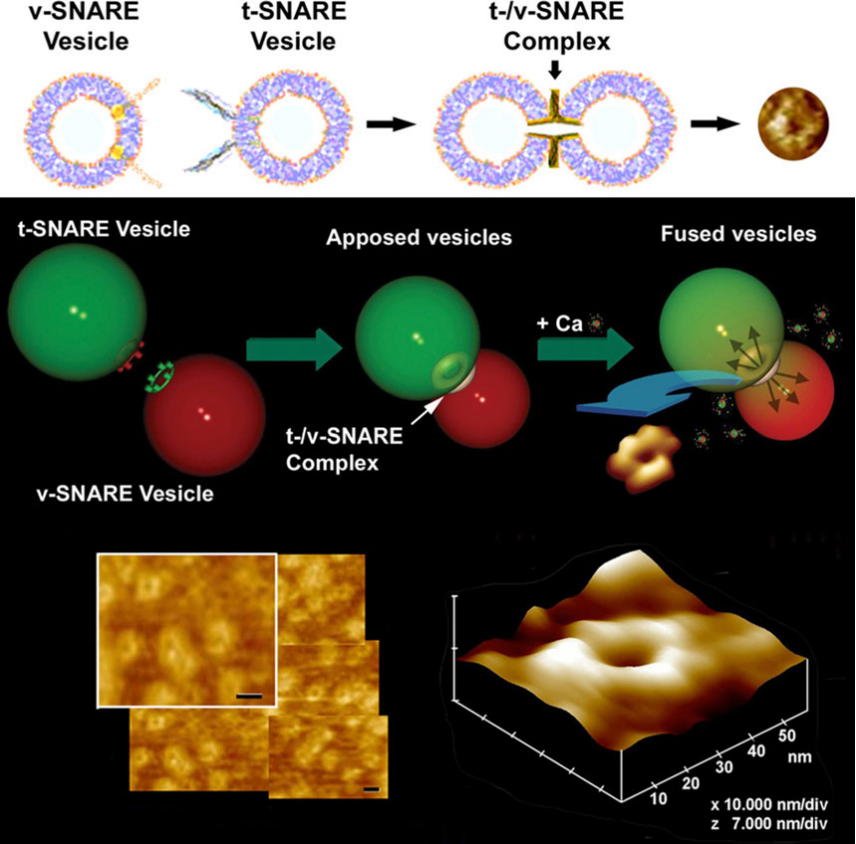
Actual t-/v-SNARE ring complexes or rosettes formed following the interaction between t-SNAREs and v-SNAREs present in opposing bilayers. Top panel is a schematic drawing depicting the interaction between t-SNAREs and v-SNAREs in opposing vesicles. AFM micrographs of the actual SNARE complex rings or rosettes are presented in the top panel (extreme right); in the middle panel (lower right); and in the lower panel at low and high resolution 21,22. These t-/v-SNARE ring complexes are obtained using large vesicles; however, 50 nm lipid vesicles associated with t-SNAREs and similar sized vesicles with v-SNAREs interact to form 6–7 nm in diameter t-/v-SNARE rosettes. The size of the SNARE rosette formed is directly proportional to the vesicle size hence curvature is critical for the SNARE complex assembly size 24,29. ©Bhanu Jena.

To address this issue, we carried out high-resolution AFM studies combined with electrophysiological measurements. In a study reported in the Biophysical Journal in 2002 21, our group demonstrated that in the absence of membrane association, SNAREs fail to appropriately bind to each other or establish continuity between the opposing bilayers in presence of calcium 21. We demonstrated that VAMP-1 proteins present in one membrane interact with syntaxin and SNAP-25 proteins present in an opposing membrane, and assemble in a rosette or ring configuration, establishing continuity between the opposing bilayers in the presence of calcium. While it had been hypothesized that the interaction between t-SNAREs and v-SNARE present in opposing bilayers may form such rosette or ring structures 43, the experimental confirmation of this was first reported by our group in the 2002 in the Biophysical Journal paper 21 (Fig.9), and further established using high-resolution EM 21–27. This SNARE rosette arrangement between opposing bilayers during membrane fusion is now widely accepted and published as the fundamental structure of the t-/v-SNARE complex associated with membrane fusion and cell secretion 44,45.

#### Role of Ca^2+^ in membrane fusion

In the 1970s, the late Demetrious Papahadjopoulos had proposed the involvement of inter-membrane Ca^2+^-phospholipid complex in the fusion of opposing lipid membranes 46. To determine the involvement of Ca^2+^ in membrane fusion at the atomic level, we performed X-ray diffraction studies involving t- and v-SNARE reconstituted liposomes 22. Results from this study demonstrated that SNAREs overcome the repulsive forces between the opposing negatively charged lipid membranes to bring them within a distance of 2.8 Å 22. We therefore concluded that if calcium was involved in the bridging of opposing bilayers *via* oxygens of the phospholipid head groups, calcium must be present at the site where the t-SNARE vesicles and v-SNARE vesicles make contact. t-SNARE vesicles and v-SNARE vesicles complexed in the absence of calcium would therefore fail to establish continuity between the opposing bilayers as hydrated calcium (with six water molecules surrounding it) measuring nearly 7 Å would be unable to fit within the 2.8 Å spacing separating the two opposing membranes. In 2004, this hypothesis was tested and confirmed experimentally by us 23. From these results, we further hypothesized that following bridging of the opposing phospholipids by hydrated Ca^2+^, the loss of coordinated water associated with the calcium ion as well as those associated with the oxygens of the phospholipid head groups must result in local dehydration, lipid mixing and membrane fusion. We tested this hypothesis using blind molecular dynamic simulations involving dimethyl phosphate (DMP), calcium and water molecules 47. Confirming this hypothesis, results from the study demonstrated that hydrated Ca^2+^ is capable of bridging phospholipid head groups, and that this process results in the expulsion of water from both phospholipid head groups and the calcium ion 47. The simulation further demonstrated that the distance between the anionic oxygens in DMP bridged by calcium is 2.92 Å, which is in close agreement with the 2.8 Å reported from X-ray diffraction measurements 22,23. These findings provide new insights into our understanding of the chemistry of membrane fusion.

### Regulation of secretory vesicle volume increase and its requirement for fractional release of intra-vesicular contents from cells during secretion

**Figure 10:**
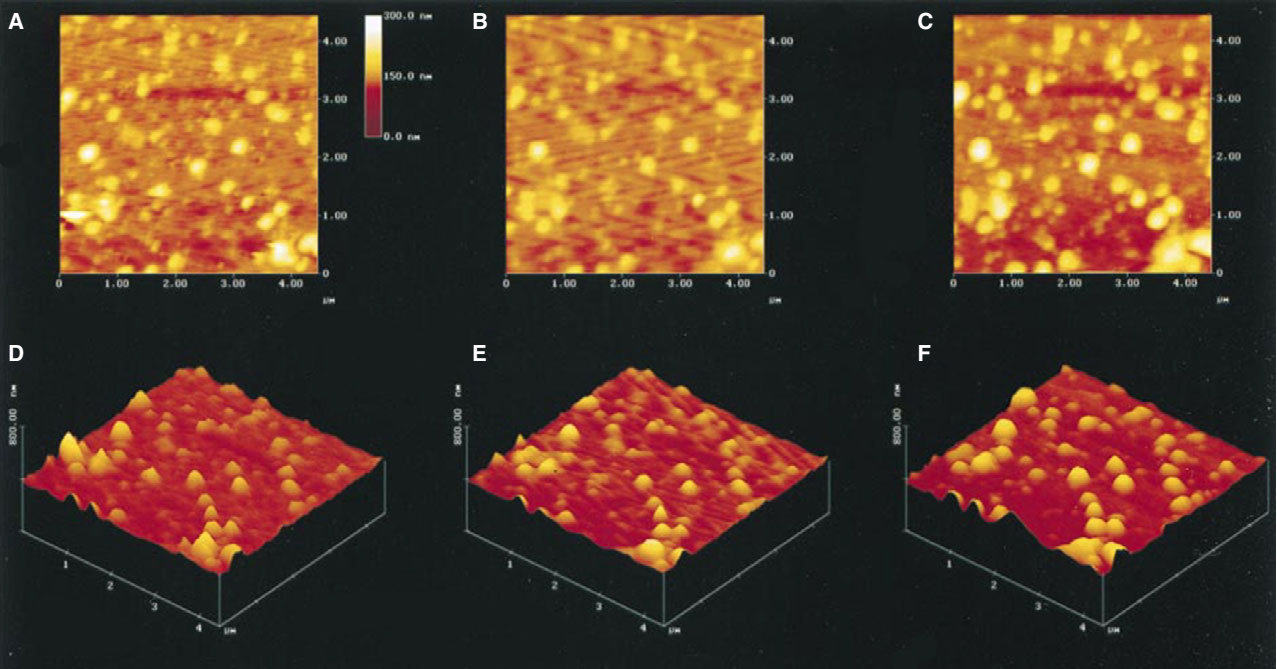
Increase in size of isolated ZGs in the presence of GTP. (A–C) Two-dimensional AFM images of the same granules following exposure to 20 μM GTP at time 0 (A), 5 min. (B) and 10 min. (C). (D–F) The same granules are shown in 3D: the three-dimensional image of the ZGs at time 0, 5 and 10 min., respectively, after exposure to GTP 30. ©Bhanu Jena.

In the early 1990s, it was reported that secretory vesicles undergo an increase in volume during cell secretion 48,49. However, the molecular mechanism underlying volume regulation of secretory vesicles and the role of this volume increase on secretory vesicle function during cell secretion was poorly understood. Our studies showed that water channels or aquaporins in conjunction with several ion channels present at the secretory vesicle membrane regulate the vesicle volume through Guanosine diphosphate(GTP)-binding G-proteins (Figs10 and 1) 30–36. The role of various ion channels at the secretory vesicle membrane was also demonstrated using single vesicle patch studies 50. In 2004, we reported that secretory vesicle volume increase is a requirement for the regulated release of vesicular contents from cells (Fig.2) 32. The relative increase in vesicle volume during cell secretion is proportional to the fraction of the intra-vesicular contents released.

**Figure 11:**
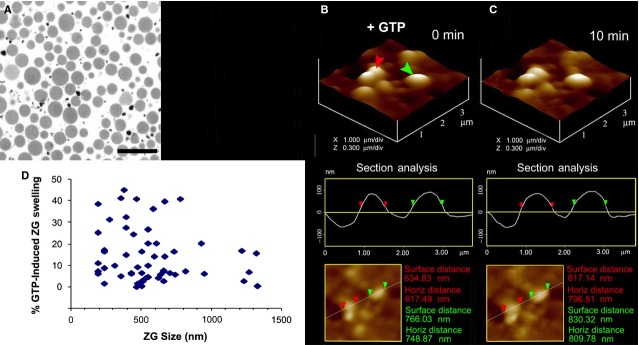
Differential swelling of isolated ZGs following GTP exposure. (A) Electron micrograph of isolated ZGs demonstrating a pure preparation. The black electron-dense particles represent inert percoll used in the ZG isolation; bar = 2.5 μm. (B and C) Isolated ZGs, on exposure to 20 μM GTP, swell rapidly. As an example, the enlargement of ZGs as determined by AFM section analysis of two vesicles (red and green arrowheads), is presented. (D) Percent ZG volume increase in response to 20 μM GTP. Note how different ZGs respond to the GTP-induced swelling differently 32. ©Bhanu Jena.

**Figure 12:**
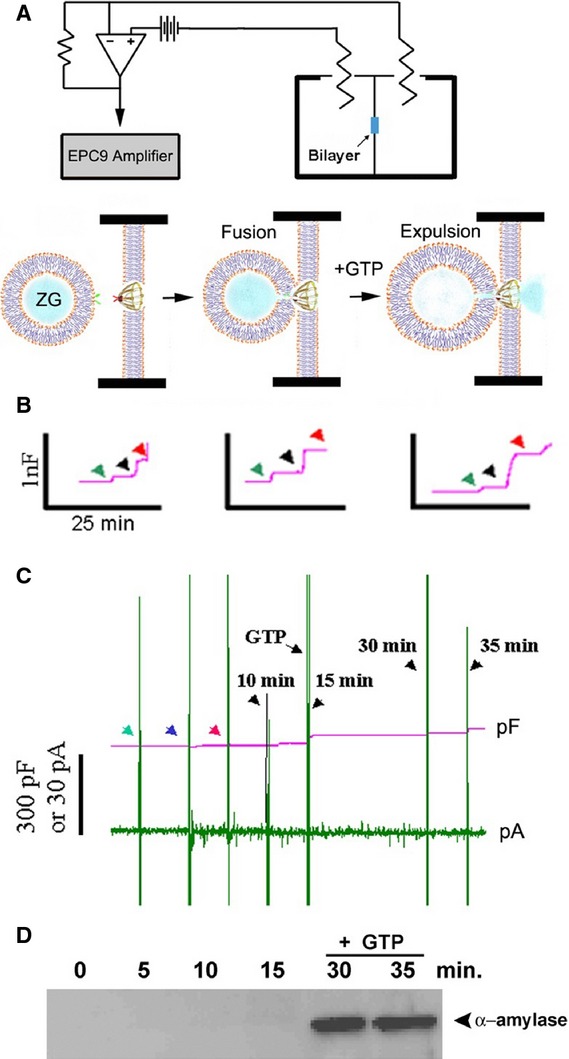
Fusion of isolated ZGs at porosome-reconstituted bilayer and GTP-induced expulsion of α-amylase. (A) Schematic diagram of the EPC9 bilayer apparatus showing the cis and trans compartment. Isolated ZGs when added to the cis compartment fuse at the bilayers-reconstituted porosome. Addition of GTP to the cis chamber induces ZG swelling and expulsion of its contents such as α-amylase to the trans bilayers chamber. (B) Capacitance traces of the lipid bilayer from three separate experiments following reconstitution of porosomes (green arrowhead), addition of ZGs to the cis compartment (blue arrowhead), and the red arrowhead represents the 5 min. time-point after ZG addition. Note the small increase in membrane capacitance following porosome reconstitution, and a greater increase when ZGs fuse at the bilayers. (C) In a separate experiment, 15 min. after addition of ZGs to the cis compartment, 20 μM GTP was introduced. Note the increase in capacitance, demonstrating potentiation of ZG fusion. Flickers in current trace represent current activity. (D) Immunoblot analysis of α-amylase in the trans chamber fluid at different times following exposure to ZGs and GTP. Note the undetectable levels of α-amylase even up to 15 min. following ZG fusion at the bilayer. However, following exposure to GTP, significant amounts of α-amylase from within ZGs were expelled into the trans bilayers chamber 32. ©Bhanu Jena.

#### Molecular underpinnings of volume regulation in secretory vesicles

Isolated secretory vesicles, single vesicle patch and reconstituted swelling-competent proteoliposomes have been utilized 30,31,33–36,50 to determine the mechanism and regulation of vesicle swelling. Isolated ZGs from the exocrine pancreas swell rapidly in response to GTP 30,31, suggesting rapid water gating into ZGs. Results from studies demonstrate the presence of the water channel aquaporin-1 (AQP1) at the ZG membrane 31 and AQP6 at the synaptic vesicle membrane 33 and their participation in GTP-mediated water entry and vesicle swelling. Further, the molecular regulation of AQP1 at the ZG membrane has been studied 34, providing a general mechanism of secretory vesicle swelling. Detergent-solubilized ZGs immunoisolated using monoclonal AQP-1 antibody, co-isolates AQP1, Phospholipase A2 (PLA), G_αi3_, potassium channel IRK-8 and the chloride channel ClC-2 34. Exposure of ZGs to either the potassium channel blocker glyburide or the PLA2 inhibitor ONO-RS-082 blocks GTP-induced ZG swelling. Red blood cells known to possess AQP1 at the plasma membrane also swell on exposure to the G_αI_ agonist mastoparan and responds similarly to ONO-RS-082 and glyburide, as do ZGs 34. Artificial liposomes reconstituted with the AQP1 immunoisolated complex from solubilized ZG preparation also swell in response to GTP. Glyburide or ONO-RS-082 is found to abrogate the GTP effect in reconstituted liposomes. AQP1 immunoisolate-reconstituted planar lipid membrane demonstrate conductance which is sensitive to glyburide and an AQP1-specific antibody. These results demonstrate a G_αi3_–PLA2-mediated pathway and potassium channel involvement in AQP1 regulation at the ZG membrane 34, contributing to ZG swelling. Similarly, AQP-6 involvement has been demonstrated in GTP-induced and G_o_-mediated synaptic vesicle swelling in neurons 33.

To further characterize the ion channels present at the secretory vesicle membrane, for the first time studies were carried out using single ZG patch 50. These studies confirm earlier findings of the presence of both potassium and chloride ion channels at the ZG membrane. In these studies, the electrical activity at the ZG membrane displays a range of sensitivity both to chloride and potassium channel blockers. Whole vesicle conductance was decreased with the addition of the chloride channel blocker, 4,4’-Diisothiocyano-2,2’-stilbenedisulfonic acid, and the Adenosine triphosphate(ATP) K^+^ channel blocker, glyburide, in both vesicles patches and indirect analysis, supporting the hypothesis for the presence of more than one channel type 50. This finding was further confirmed immunochemically using Western blot analysis, and as speculated, the presence of two chloride channels, CLC-2 and CLC-3, was observed 50. Also consistent with pharmacological evidence was the presence of ATP-sensitive potassium channel, Kir6.1 in Western blot analysis of the ZGs. This is surprising, as Kir6.2 is the predominant form of potassium channels in β cells of the endocrine pancreas 50.

As mastoparan, an amphiphilic tetradecapeptide from wasp venom, activates G_o_ protein GTPase and stimulates synaptic vesicle swelling, the presence of β-adrenergic receptor at the synaptic vesicle membrane was hypothesized. Stimulation of G-proteins is believed to occur *via* insertion of mastoparan into the phospholipid membrane to form a highly structured α-helix that resembles the intracellular loops of G-protein-coupled adrenergic receptors. Consequently, the presence of adrenoceptors and the presence of an endogenous β-adrenergic agonist at the synaptic vesicle membrane has been investigated. Immunoblot analysis of synaptic vesicle using β-adrenergic receptor antibody, and vesicle swelling experiments using β-adrenergic agonists and antagonists demonstrate the presence of functional β-adrenergic receptors at the synaptic vesicle membrane 36.

In summary, our studies in the past two decades demonstrate the presence of a new cup-shaped lipoprotein structure at the cell plasma membrane called ‘porosome – the universal secretory portals in cells’, and elucidates how the porosome is involved in the regulated fractional release of intra-vesicular contents from cells with exquisite precision involving membrane fusion and secretory vesicle volume regulation, revealing for the first time the molecular underpinnings of the transient or kiss-and-run mechanism of secretion in cells. We have isolated the porosome from a number of secretory cells including neurons, determined its composition, functionally reconstituted it in lipid membrane, and determined its dynamics and high-resolution structure using a variety of approaches including AFM, EM and SAXS. Complementing the regulation of the porosome function, our studies have further contributed to our understanding of SNARE and Ca^2+^-mediated membrane fusion and secretory vesicle volume regulation, both required for the regulated fractional release of intra-vesicular contents during cell secretion. These results provide for the first time a molecular understanding of the regulated fractional release of intra-vesicular contents from cells during secretion. Recent studies by our group using mass spectrometry, demonstrate interaction between the cystic fibrosis trans-membrane conductance regulator (CFTR) and the porosome complex in human airways epithelia, shedding light on the possible regulatory role of CFTR on the quality of mucus secretion *via* the porosome complex 51. Results from this study provide critical insights into the aetiology of CF disease and for potential therapies. Similarly, recent studies using mass spectrometry provide some understanding of the lipidome of the neuronal porosome complex 38, and the role of Hsp90 in porosome assembly and function 52, enabling further understanding of the porosome structure–function 37. Currently, for further understand porosome structure–function, the major focus of our laboratory is to determine the distribution and interaction of proteins and lipids within the porosome complex using chemical crosslinking followed by mass spectrometry, and molecular modelling. To gain further structural insights especially on the neuronal porosome complex (as it is the smallest of the known porosomes, abundantly present and has been most extensively studies) single particle cryo-electron tomography, SAXS and neutron scattering, and other associated approaches are being utilized.
